# Skin Manifestations and Coeliac Disease in Paediatric Population

**DOI:** 10.3390/nu13103611

**Published:** 2021-10-15

**Authors:** Flavia Persechino, Gloria Galli, Severino Persechino, Francesco Valitutti, Letizia Zenzeri, Angela Mauro, Vito Domenico Corleto, Pasquale Parisi, Chiara Ziparo, Melania Evangelisti, Giovanna Quatrale, Giovanni Di Nardo

**Affiliations:** 1 Department of Clinical and Molecular Medicine, Sapienza University of Rome, 00185 Rome, Italy; flaviapersechino@hotmail.it; 2 Department of Medical-Surgical and Translational Medicine, Sant’Andrea University Hospital, Sapienza University of Rome, 00185 Rome, Italy; gloria.galli5@gmail.com (G.G.); vito.corleto@gmail.com (V.D.C.); 3 Dermatology Unit, NESMOS Department, Faculty of Medicine & Psychology, Sapienza University of Rome, Sant’Andrea University Hospital, 00185 Rome, Italy; Severino.persechino@uniroma1.it; 4 Pediatric Unit, AOU San Giovanni di Dio e Ruggi D’Aragona, Salerno, Italy and EBRIS (European Biomedical Research Institute of Salerno), 84121 Salerno, Italy; francesco.valitutti@gmail.com; 5 Pediatric Emergency Unit, Emergency Pediatric Department, AORN Santobono-Pausilipon Children’s Hospital, 80129 Naples, Italy; zenzeriletizia@gmail.com (L.Z.); angela.mauro84@gmail.com (A.M.); 6 Pediatric Unit, NESMOS Department, Faculty of Medicine & Psychology, Sapienza University of Rome, Sant’Andrea University Hospital, 00185 Rome, Italy; pasquale.parisi@uniroma1.it (P.P.); chiaraziparo@gmail.com (C.Z.); melania.evangelisti@uniroma1.it (M.E.); giovanna.quatrale@gmaill.com (G.Q.)

**Keywords:** coeliac disease, skin manifestation, children

## Abstract

Celiac disease (CD) is an immune-mediated enteropathy caused by gluten ingestion, affecting approximately 1% of the worldwide population. Extraintestinal symptoms may be present as the first signs of CD, years before the CD diagnosis is made. A great variety of extraintestinal manifestations may be associated with CD. Cutaneous manifestations represent the main extraintestinal manifestations, with dermatitis herpetiformis being the most common in patients with CD. In adults, it has been demonstrated that the role of a gluten-free diet is crucial not only for the recovery of signs and symptoms associated with CD but also for cutaneous manifestations, which often improve after gluten avoidance. In children with CD, the association with skin disorders is well documented regarding dermatitis herpetiformis, but studies considering other dermatological conditions, such as psoriasis and atopic dermatitis, are few. The prevalence and manifestations of dermatological disorders in celiac children are often different from those in adults, explaining the gap between these populations. In addition, the therapeutic role of a gluten-free diet in the improvement in skin alterations is not fully understood in children and in adult population except for dermatitis herpetiformis. Therefore, cutaneous CD symptoms need to be known and recognized by physicians despite their specialties to improve early CD diagnosis, which is critical for a better prognosis. This review describes the current scientific evidence on skin manifestations associated with CD in the pediatric population.

## 1. Introduction

Celiac disease (CD) is a systemic immune-mediated condition characterized by an aberrant response to wheat gliadins and other cereal prolamins that causes small intestinal enteropathy and a wide range of symptoms in genetically susceptible individuals [1]. In the pediatric population, the actual prevalence of the disease in many Western countries ranges from 0.10% to 3.03%, with a significantly increasing annual trend [2]. Concomitant with the increase in CD incidence, changes in its clinical presentation have been described in the past few decades. A higher number of asymptomatic cases together with an increase in the number of “non classical” presentations, including extraintestinal symptoms such as iron deficiency anemia, altered bone metabolism, short stature, elevated liver enzymes and skin manifestations, have been detected by targeted screening of at-risk groups [3]. A recent study showed that non intestinal symptoms were the most commonly represented symptom in 43% of pediatric CD patients [4]. Nevertheless, the clinical presentation of the disease can often be misleading as highly variable from one patient to another, leading to frequent delays in diagnosis [1]. Therefore, extraintestinal CD needs to be recognized by physicians of various specialties, including gastroenterologists, internists, pediatricians, neurologists, dermatologists, gynecologists, and particularly general practitioners. 

CD patients also present an increased risk for autoimmune disorders compared to healthy controls, with a prevalence ranging from 14% to 27% [5,6]. In addition, a higher prevalence of CD has been recognized in patients with a number of autoimmune disorders, warranting however an active screening only in a few diseases, such as type I diabetes mellitus [7]. There are some hypotheses related to this association: the shared genetic features between CD and the other immune-mediated disorders, the alteration of the intestinal barrier, and a similar environment trigger [8]. The loss of the intestinal barrier permeability could be related to both local and systemic inflammation triggered by lymphocytes T activation induced by gluten with the subsequent release of proinflammatory cytokines [9]. Even if not yet fully understand, the beneficial effect of the start of GFD observed in some concomitant autoimmune disorders could be partially secondary to the end of this immunity process and the consequent reduction in the release of the proinflammatory cytokines trigger [10].

Among extraintestinal CD manifestations, there are many cutaneous diseases. Skin diseases associated with CD have been classified into those improved by a gluten-free diet (GFD) and those occasionally associated with CD [11]. The risk to develop skin disorders is higher at the CD diagnosis but persists beyond 10 years [12]. 

In this review, the main features of skin diseases with a proven association with CD and those improving after a GFD are described in children. Moreover, other pediatric skin conditions sporadically associated with CD are briefly reported. In particular, we aim to improve the early recognition of CD-associated skin manifestations, recommending active case finding for CD and thus reducing the risk of diagnostic delay.

## 2. Dermatitis Herpetiformis

Dermatitis herpetiformis (DH) is a chronic, itchy, gluten-triggered skin disorder whose features are subepidermal granular IgA deposits leading mainly to papulovesicular lesions [13,14].

Links between CD and DH have emerged in recent decades: the disappearance of the skin rash on a GFD, the occurrence of both conditions within families and the overlapping human leukocyte antigen (HLA) [15,16,17]. Approximately 85% of DH patients with a Caucasian background carry HLA-DQ2, while most others carry HLA-DQ8 [18]. Even though DH is not uncommon among adolescents, it is rarely observed before puberty, with most children being diagnosed between the ages of 2 and 7 years [19,20].

DH is most prevalent in individuals of Northern European descent. In adults, men with DH slightly outnumber women by nearly 1.5:1 [21,22,23]; nevertheless, there is a female predominance in the pediatric population, and it seems more prevalent in Mediterranean areas [24,25]. At present, the DH-to-CD prevalence is 1:8 [26].

**Figure 1:**
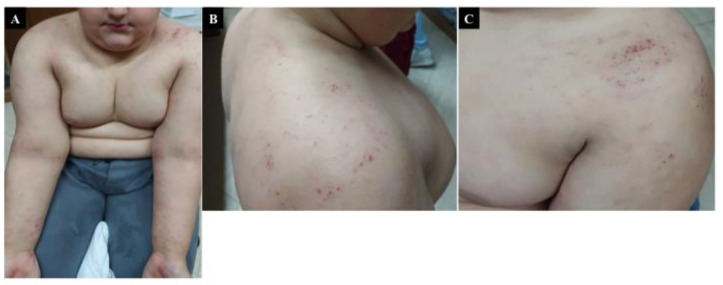
Dermatitis Herpetiformis. (**A**) Erythematous, papular, and vesiculosus lesions in a 14 year old child with Atopic Dermatitis and diagnosis of Celiac disease. (**B**,**C**) a magnification of the DH shows a typical polymorphism consisting of erythema, urticarial plaques, papules, grouped vesicles and blisters associated with intense itch and therefore followed by erosions, excoriations, and hyperpigmentation.

The clinical picture of DH is characterized by papulovesicular lesions and urticarial plaques, which are intensively itchy and symmetrically distributed, typically affecting the extensor surfaces (elbows, knees, and buttocks) [26]. As the lesions are strongly pruritic, typical vesicles are rarely apparent since they turn into excoriation (Figure 1). DH presents similarly in children and adults; however, children might have uncommon skin findings, such as solitary involvement hemorrhagic lesions of the palms and soles, as well as deep dermal papules/nodules and facial lesions [27,28].

Acral purpura and petechiae are a common finding among DH patients and may even indicate an early-stage disease: these hemorrhagic skin lesions affect the fingers and the toes primarily and are referred to as digital purpura [29].

In this case, 25% of DH patients have normal small bowel villous architecture or minor changes in terms of increased intraepithelial lymphocytosis [30,31]. However, whether villous atrophy is present in DH patients does not affect the long-term prognosis of skin lesions [31].

Instead, DH is now a common extraintestinal manifestation of CD occurring in up to 10% of CD patient series in Europe and North America, but it has also been described more recently in other geographical areas [32,33]. DH patients rarely complain of gastrointestinal symptoms, albeit sometimes these subjects can remain unaware of those before diagnosis [34].

Intriguingly, features of DH and CD (without visible skin lesions) can alternate in the same person or their family members during different periods of life, thus tightly embedding the two diseases within the same spectrum [35,36].

Direct immunofluorescence (DIF) microscopy is the gold-standard procedure for DH diagnosis; however, it should be noted that false-negative immunofluorescence results occur in approximately 5% of cases, especially if blisters or inflamed skin areas have been biopsied. The key elements for diagnosing DH at DIF are granular IgA deposits in the papillary dermis of peri-inflamed areas [37]. IgA granular deposits may persist even decades after commencing a strict GFD [38]; thus, in doubtful cases, DH diagnosis can also be ratified afterwards, without necessarily reintroducing gluten.

Conventional histopathological examination of DH lesions shows neutrophilic micro-abscesses in the dermal papillae with or without subepidermal blisters [39]. Nevertheless, these findings are not fully specific to DH, as similarities can be detected in other blistering skin diseases [40]. Therefore, it is still under scrutiny whether a biopsy for conventional histology is mandatory since granular IgA deposits at DIF, together with a compatible clinical picture, might suffice to confirm DH. However, a European consensus statement among experts suggests that a 4–5 mm punch biopsy of the lesion should be taken regardless, particularly to address differential diagnoses [13] of other vesiculobullous disorders such as linear IgA disease, pemphigoid, eczema, and scabies [41].

Serum IgA-class antibodies against TG2, the autoantigen of CD, frequently circulate in undiagnosed patients with DH and should always be kept in mind in clinical practice [42]. IgA-class antibodies against TG3, the autoantigen of DH, are measurable in the serum of several patients with DH and a smaller percentage of those with CD [43]. However, the specificity of serum TG3 antibody assessment for DH and CD is currently unknown. As a consequence, TG3 antibodies are still reserved for research purposes only.

DH management is similar for children and adults. Ideally, treatment consists of a GFD with a resolution of cutaneous symptoms in 1–6 months in about 80% of patients [20]. Thus, if symptoms persist despite a strict GFD or there is only a reduction of the rush, dapsone (2 mg/kg/day or 4 mg/kg weekly) might be considered an add-on therapy [20,44].

As CD and DH are strictly embraced in suspected DH lesions, serological screening with IgA anti-transglutaminase, EMA and total IgA is necessary in order to establish both the DH and the CD diagnosis. IgA anti-transglutaminase evaluation is useful also for the monitoring of GFD adherence [13]. 

## 3. Psoriasis

Psoriasis is one of the most common chronic immune-mediated inflammatory disorders affecting approximately 2% of the global population [45,46]; one-third of cases occur in children [47] with a mean age of onset of 8 to 11 years [48,49].

Psoriatic skin lesions are characterized by erythematous scaly plaques tending to have a relapsing and remitting course. In childhood, the disease onset often occurs as a guttate form, evolving in plaque psoriasis in one-third of these patients [45]. Typical erythematous plaques with overlying white scales are often thinner and smaller than adults’ and tend to develop more often on the face and flexural areas, even if potentially present on the whole-body surface [50]. Many comorbidities, such as obesity, metabolic syndrome, arthritis, Crohn’s disease, uveitis, and diabetes, have been described in children with psoriasis [51].

The prevalence of CD in psoriatic adult patients is between 0.3 and 14.6% [52,53,54]. This wide percentage is mainly due to the diagnostic strategy considered to rule out CD, based only on antibody positivity or on duodenal histological evaluation. Two meta-analyses in an adult population showed that psoriatic patients were more than twice as likely to have a coexisting diagnosis of CD [55,56]. Conversely, it has been found that CD patients were approximately 1.8-fold more likely to have psoriasis [53], with a persisting risk even 5 years after CD diagnosis [57]. Some other studies did not find evidence of an association between psoriasis and CD markers, but these were small-size studies, and some lacked control groups [58,59,60,61].

Studies focused only on pediatric CD populations are scant. Thus, the prevalence of psoriasis in children with CD is currently uncertain. A study conducted in an adult population including children with CD also showed a positive association between these two diseases with a hazard ratio of 2.05 (CI 1.62–2.60) to develop psoriasis before and after CD diagnosis [57]. Other studies, including both children and adults, reported an increased association between CD and psoriasis [62,63]. A recent study of CD in adolescents demonstrated a consistent association with psoriatic skin disorder with a relative risk of 1.6 (CI 1.1–2.2) to develop this cutaneous disease [64]. In contrast, a recent retrospective study performed on 1925 pediatric patients with psoriasis did not find a significant association between CD and psoriasis [65]. Due to these scant data, the current pediatric psoriasis comorbidity guidelines do not support mandatory CD screening in all children with psoriasis [66].

Few studies have examined the role of the GFD in psoriasis manifestations leading to heterogeneous results. Studies evaluating patients with AGA (anti-gliadin antibodies) positivity, even without a defined CD diagnosis, reported a significant improvement in the PASI (psoriasis area and severity index) after a GFD compared to those with AGA negativity [67,68]. One study has assessed the effect of a GFD on psoriatic manifestations in patients with biopsy-proven CD, reporting a significant 6-months persistent improvement of the PASI in 9/10 patients after starting the GFD [69]. However, this improvement was not compared with the control group. A favorable role of a GFD was also reported in a case report [70]. 

To our knowledge, no studies have analyzed the role of the GFD exclusively in children with psoriasis. An isolated case report described a 5-year-old child with psoriasis and a subsequent diagnosis of CD, reporting only a slight improvement in the skin lesions after a GFD [71]. 

In summary, an active case finding with a shallow threshold to test should also be applied among psoriatic patients. In addition, the GFD may potentially be useful in psoriasis patients with a diagnosis of CD or with CD-specific antibodies positivity, but more well-conducted studies are needed. 

## 4. Alopecia Areata

**Figure 2:**
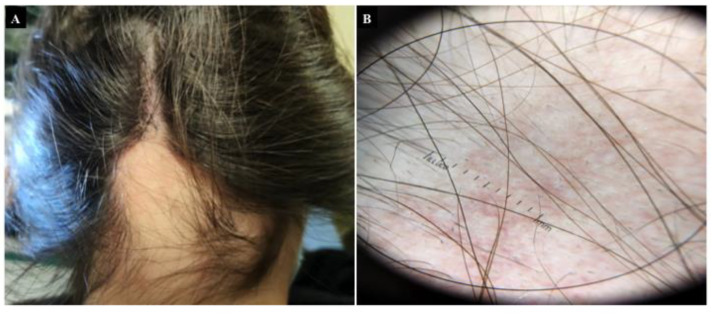
Alopecia Areata. (**A**) nonscarring hair loss in a 16 year old woman with onset diagnosis of Celiac Disease. (**B**) Dermoscopic image with a magnification 20×: dermoscopic features in alopecia areata are black dots (cadaverous hairs), yellow dots, tapering hairs (exclamation mark hairs), and broken hairs.

Alopecia areata (AA) is a patchy, nonscarring hair loss of the scalp that affects approximately 2% of the global population [72] and has a slightly higher prevalence in children and adolescents; 66% of patients with AA are younger than 30 years of age [73]. The sex distribution was approximately equal (M:F = 1:1.1). Diagnosis is clinical in all cases, based on a typical history of abrupt, patchy loss of hair, with or without progression, and a normal-looking scalp, without any secondary characteristics on examination (Figure 2) [73,74]. Psychological stress and anxiety have been reported to play an essential role in the precipitation and exacerbation of AA, and in contrast, AA is often a trigger for symptoms of depression and anxiety [75,76].

It has been estimated that approximately 7–14% of adult patients present more severe forms progressing to the total loss of scalp hair (*alopecia totalis—AT*), eventually associated with the loss of whole-body hair (*alopecia universalis—AU*) [77,78]. Childhood-onset is a negative prognostic factor as it is associated with a more severe disease with poor hair regrowth [73,79]. Nail’s alterations (pitting, striations, and brittleness) are also described in about 30% of patients [80].

The etiopathogenesis of AA is still unclear, even if an autoimmune T-cell-mediated reaction to the hair follicle has been recognized [81,82]. Other autoimmune disorders have been demonstrated in patients with AA, such as Addison’s disease, autoimmune thyroiditis, atrophic gastritis, systemic lupus erythematosus, rheumatoid arthritis, and vitiligo [81]. 

Studies performed in children on its association with CD demonstrated that a percentage from 0.7% to 2% of CD patients had AA, a percentage similar to that found in the general population [83,84]. Two studies carried out at the end of the 1990s on both adults and children with AA showed an estimated prevalence of biopsy-proven CD between 1:116 and 1:85, higher respect to the CD prevalence reported at that time in the general population (1:305), but in line with the actual disease prevalence [85,86]. One more recent study conducted on a small population (35 patients), considering only antibodies positivity without performing histological duodenal sampling, reported a prevalence of CD of 2.9% [87]. These data fail to fully demonstrate a higher risk of CD development in individuals with AA. 

In CD patients, the role of the GFD in the improvement of AA lesions is controversial. Evidence is limited and mainly based on case reports or studies conducted with a small number of patients and without a control group. Considering a total of 31 patients, both adult and children, with concomitant CD and AA diagnosis reported in different studies, after the start of the GFD 70.9% presented hair regrowth (both partial and complete), 22.6% presented no regrowth, and 6.5% had no compliance with the diet [88]. In these patients, the response to the GFD has been assessed between 6–24 months, but most patients reported an improvement after a shorter time [85,86,89,90,91,92]. In one case series and one case report of patients with a CD diagnosis, a very limited effect of GFD in hair regrowth has been described, above all in those with more severe and extensive hair alterations (AT or AU) [91,92]. It should also be highlighted that AA lesions present a high rate of spontaneous remission [73], therefore the GFD role in hair regrowth should be confirmed in wider and well-conducted studies.

Concerning serological screening for CD in AA patients, according to the discussed data, it could be suggested in those patients with other risk factors or clinical/biochemical suspicion for CD.

## 5. Chronic Urticaria

Urticaria is a common systemic disease, occurring in 15–25% of individuals [93]. Clinically, we observe pink-to-red oedematous, itchy lesions that often have pale centers, can range in size from a few millimeters to several centimeters in diameter and are often transient, lasting for less than 48 h. Approximately 40% of patients with urticaria also experience angioedema [94]. Chronic urticaria (CU) occurs when the lesions occur for more than 6 weeks [95]. CU is often associated with significant morbidity and poor quality of life [96].

Hauteke et al. described the association between CD and CU for the first time, although the relationship between these two diseases is not fully clear [97]. Caminiti et al. performed a case–control study to determine the occurrence of CD in urticaria and matched control children. They found that CD was significantly more frequent in children with CU than in controls and that a GFD resulted in urticaria remission. Thus, CD may be regarded in such subjects as a cause of CU [98]. Ludvigsson and colleagues examined the association between CD and urticaria in the largest population-based cohort study involving 28,900 patients with biopsy-verified CD (both adults and children). They found a 1.5-fold increased risk of urticaria in CD, with a slightly higher risk for chronic urticaria (HR = 1.92). Furthermore, patients were at a moderately increased risk of receiving a diagnosis of urticaria or chronic urticaria before CD diagnosis [99].

The autoimmunity induced by gliadin or by other unknown antigens may link CU and CD. Levine A et al. proposed that the increased permeability of the intestinal mucosa allows the passage of antigens responsible for CU pathogenesis by the formation of circulating immunocomplexes [100]. Theoretically, this mechanism might cause urticarial lesions, so by restoring the integrity of the mucosa, a GFD might resolve them [101]. Furthermore, even though no meta-analysis is available, in some cases of CU, the adoption of a GFD has proven effective in controlling skin lesions in CD patients, further confirming that CU may be a cutaneous manifestation of CD and not a mere chance association [98,102]. Recently, Kolkhir et al. stated that CU is strongly linked with various autoimmune diseases, such as Hashimoto’s thyroiditis, pernicious anemia, vitiligo, diabetes mellitus type 1, Grave’s disease, and rheumatoid arthritis, and that it is essential to test children with chronic spontaneous urticaria (CSU) for other autoimmune diseases, including CD [103]. In addition, CD can be diagnosed in children with CSU even in the absence of other features; when starting a GFD, the decrease in the immunologic stimulus may account for symptom improvement in children with subclinical CD associated with CU [104]. Even though limited evidence is available, a practical recommendation to screen for CD in chronic unexplained urticaria sounds reasonable.

## 6. Atopic Dermatitis

**Figure 3:**
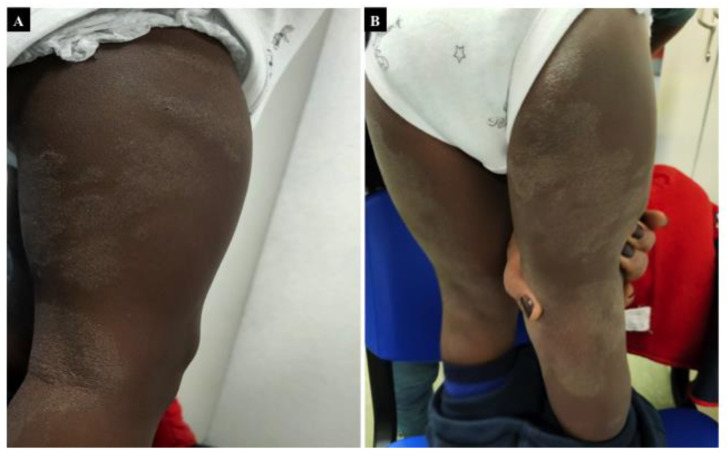
Atopic Dermatitis. (**A**,**B**) erythema, lichenification, scaling, and prurigo in a 6 years old child. Excoriated bilateral erythematous scaling papules and plaques on the surface of the lower limbs.

Atopic dermatitis (AD) is a chronic inflammatory dermatosis that affects approximately 15–20% of children (Figure 3) [105,106]. It affects the pediatric population beginning in early childhood, and it may be subsequently associated with symptoms typical of other allergy diseases, such as food allergies [107]. Pediatricians generally manage AD, and it can affect children with a different degree of severity (the disease is mild in 76.4%) [108]. Most AD patients present a positive family history of atopic diseases, suggesting a genetic predisposition, and have food or aero-allergen sensitization [109].

Recent studies have demonstrated that AD patients are at risk of several autoimmune diseases [110]. In the literature, few studies have investigated the relationship between CD and AD in children, and the results are controversial.

The first study performed in a sample of 42 pediatric patients with CD found a higher prevalence of AD than that in a healthy pediatric population (45% vs. 25%, respectively) [111]. In contrast, Greco et al., in a sample of 83 patients affected by CD, showed that there was no increase in the rate of atopic diseases in CD patients compared to healthy pediatric controls [112]. Ress et al. showed a CD prevalence of 1.4% in a sample of 351 pediatric patients with AD with a four-times higher risk of being affected by CD (odds ratio 4.18; 95% confidence interval 1.1–15.7) [113].

Likewise, in one of the largest cohort studies (9290 adult and 10,196 pediatric AD patients), a statistically significantly higher risk of CD was not observed in children and adolescents with AD (OR 2.90, 95% CI 0.88–9.54) [104]. These data were not confirmed in the most recent study performed on 71,659 pediatric patients with AD in which atopic dermatitis was associated with a significantly higher prevalence of CD in the multivariate analysis (OR 1.609; 95% CI 1.42–1.82, *p* < 0.001) [114]. In the most recent retrospective study, it has been observed a significant association between AD and CD (OR 2.28; 95% CI 2.07–2.52). [115]. To our knowledge, no data are available on the efficacy of a GFD in controlling atopic lesions in patients with CD. Despite these uncertainties, active case findings should still be encouraged for children with AD showing+ other CD-related symptoms as well as in high-risk patients for CD.

## 7. Hereditary Angioneurotic Oedema

Hereditary angioneurotic oedema (HANE) is the most common genetically linked clinical disorder caused by a protein deficiency associated with complement activation. Hereditary angioedema due to C1-INH (HAE-C1-INH) deficiency is associated with enhanced consumption of early complement components, which may predispose patients to autoimmune disease. HANE is a life-threatening condition that manifests as oedematous attacks involving subcutaneous tissues and/or the upper airway/gastrointestinal mucosa. It usually presents in late childhood or adolescence in otherwise healthy subjects, and family history is present in approximately 75% of cases [116]. Farkas et al. described for the first time the occurrence of HANE in a child with CD. An 11-year-old male had a diagnosis of CD with remission of his clinical and histologic findings. Despite adherence to a GFD and a typical appearance of duodenal biopsies, she had a monthly attack of colicky abdominal pain, vomiting and diarrhoea from the age of 14 years; attacks were sometimes accompanied by subcutaneous oedema, and the abdominal US performed during an attack showed free peritoneal fluid. A novel missense mutation was detected in the C1-INH gene, and prophylaxis with tranexamic acid prevented the recurrence of symptoms [117]. Csuka et al. screened 22 pediatric CD patients with HANE. Four out of the 22 children were diagnosed with CD, with a higher prevalence of the latter disease among pediatric patients with hereditary angioedema (22 children) than in the general population (18.1% vs. 1.2%) [118].

Interestingly, introducing a GFD mitigated only abdominal symptoms of hereditary angioedema. The authors suggested that screening hereditary angioedema patients for CD is warranted if abdominal attacks or neurological symptoms persist despite adequate management. Likewise, complement testing is recommended whenever abdominal symptoms persist despite the histological and serological remission of gluten-sensitive features after introducing a GFD [118].

## 8. Other CD-Associated Skin Conditions

CD has also been demonstrated in the pediatric population to be associated with other skin disorders. This association has been described in some case reports lacking strict evidence and significant population analysis. The other CD-associated skin conditions are reported in Table 1.

Chronic allergic vasculitis was described in a 12-year-old girl with coeliac disease. A GFD induced remission of gastrointestinal and cutaneous symptoms [119], even if the effective pathogenetic link was not demonstrated.

Vitiligo is a pigmentary skin disorder that is more frequently associated with CD in children [82]. Lupus erythematosus is a worldwide chronic autoimmune disease that may affect every organ and tissue and frequently manifests with skin involvement. It has been associated with an increased frequency of CD diagnosis [120,121,122,123]. Both of these associations could be secondary to the autoimmune pathway shared by the reported diseases.

Behcet’s disease is a type of vasculitis presenting with chronic inflammation that affects multiple systems and vessels in young patients. Behcet’s disease and CD share many similar clinical and histological manifestations, and some case reports have shown a weak association between these two diseases [124].

Further CD-associated skin conditions are reported in Table 1. 

About stomatous aphtosis, there are two pathogenetic hypotheses for these cases. The first is that stomatous aphtosis is directly influenced by gluten sensitivity, and the second is that dermatologic comorbidity is related to CD-untreated malabsorption [140,141].

Finally, CD could be associated with skin disorders due to malabsorption and deficiency of iron, folic acid, vitamin B12, zinc, vitamin A, vitamin PP, and other oligoelements.

Zinc deficiency may cause erythematous-squamous dermatitis in the periorificial regions, genitals and flexures; diffuse alopecia; and stomatitis [142].

Iron deficiency is associated with atrophic glossitis and angular stomatitis similar to vitamin B12 and folic acid deficiency [143].

## 9. Conclusions

Skin manifestations are frequently associated with CD in both adults and children. The role of GFDs is frequently crucial for the recovery of signs and symptoms associated with CD and for cutaneous manifestations as DH. GFDs might be beneficial also for some patients suffering from other skin disorders, which improve after gluten avoidance.

In contrast to adults, these associations are not entirely documented in children, and a great portion of the cutaneous manifestations are still uncertainly linked to CD.

According to this review, active case findings with CD screening should be encouraged in children with psoriasis. In patients with alopecia areata, chronic urticaria, atopic dermatitis, hereditary angioneurotic oedema, vitiligo, and other minor cutaneous disorders with limited evidence, CD screening should be reserved for children with other factors potentially relevant to CD suspicion.

## Figures and Tables

**Table 1:** Other skin condition associated to CD.

Author (Year)	Type of Study	Skin Condition
Buderus, S. [125] (1997) Molnár, K. [126] (2006)	Case reports Case reports	Dermatomyositis
Abenavoli, L. [127] (2019); Zone, J.J. [128] (2005) Bartyik, K. [129] (2004)	Review Review Case report	Erythema nodosum
Vaz, S.O. [130] (2018)	Case report	Linear IgA bullous dermatosis
Troncone, R. [131] (2014)	Review	pityriasis lichenoides
Nunnemann, S. [132] (2020)	Case report	Porphyria
Nenna, R. [133] (2011)	Case study	Ichthyosis
Howard, G. [134] (2021)	Case report	Morphea
Woolfson, H. [135] (1974) Thelander, H.E. [136] (1946)	Case report Case report	Erythroderma
Garcia, Y.H. [137] (2002) Brinkert, F. [138] (2009)	Case Report Case report	chronic mucocutaneous candidiasis
Müller, S. [139] (1999)	Case report	lypodistrophia centrifugalis abdominalis infantilis
Yılmaz, S. [140] (2020) Campisi, G. [141] (2008)	Case report Observational study	Stomatous aphtosis

## References

[1] Lebwohl B., Rubio-Tapia A. (2021). Epidemiology, presentation and diagnosis of coeliac disease. Gastroenterology.

[2] Roberts S.E., Morrison-Rees S., Thapar N., Benninga M.A., Borrelli O., Broekaert I., Dolinsek J., Martin-de-Carpi J., Mas E., Miele E. (2021). Systematic review and meta-analysis: The incidence and prevalence of paediatric coeliac disease across Europe. Aliment. Pharmacol. Ther..

[3] McGowan K.E., Castiglione D.A., Butzner J.D. (2009). The changing face of childhood celiac disease in North America: Impact of serological testing. Pediatrics.

[4] Almallouhi E., King K.S., Patel B., Wi C., Juhn Y.J., Murray J.A., Absah I. (2017). Increasing incidence and altered presentation in a population-based study of pediatric celiac disease in North America. J. Pediatr. Gastroenterol. Nutr..

[5] Ventura A., Magazzù G., Greco L. (1999). Duration of exposure to gluten and risk for autoimmune disorders in patients with celiac disease. SIGEP Study Group for Autoimmune Disorders in Celiac Disease. Gastroenterology.

[6] Conti L., Lahner E., Galli G., Esposito G., Carabotti M., Annibale B. (2018). Risk Factors Associated with the Occurrence of Autoimmune Diseases in Adult Coeliac Patients. Gastroenterol. Res. Pract..

[7] Green P.H., Cellier C. (2007). Celiac disease. N. Engl. J. Med..

[8] Parkes M., Cortes A., van Heel D.A., Brown M.A. (2013). Genetic insights into common pathways and complex relationships among immune-mediated diseases. Nat. Rev. Genet..

[9] Lundin K.E., Wijmenga C. (2015). Coeliac disease and autoimmune disease-genetic overlap and screening. Nat. Rev. Gastroenterol. Hepatol..

[10] Lerner A., Shoenfeld Y., Matthias T. (2017). Adverse effects of gluten ingestion and advantages of gluten withdrawal in nonceliac autoimmune disease. Nutr. Rev..

[11] Humbert P., Pelletier F., Dreno B., Puzenat E., Aubin F. (2006). Gluten intolerance and skin diseases. Eur. J. Dermatol..

[12] Lebwohl B., Söderling J., Roelstraete B., Lebwohl M.G., Green P.H., Ludvigsson J.F. (2020). Risk of Skin Disorders in Patients with Celiac Disease: A Population-Based Cohort Study. J. Am. Acad. Dermatol..

[13] Görög A., Antiga E., Caproni M., Cianchini G., De D., Dmochowski M., Dolinsek J., Drenovska K., Feliciani C., Hervonen K. (2021). S2k guidelines (consensus statement) for diagnosis and therapy of dermatitis herpetiformis initiated by the European Academy of Dermatology and Venereology (EADV). J. Eur. Acad. Dermatol. Venereol..

[14] Collin P., Salmi T.T., Hervonen K., Kaukinen K., Reunala T. (2017). Dermatitis herpetiformis: A cutaneous manifestation of coeliac disease. Ann. Med..

[15] Fry L., Seah P.P., Riches D.J., Hoffbrand A.V. (1973). Clearance of skin lesions in dermatitis herpetiformis after gluten withdrawal. Lancet.

[16] Reunala T., Salo O.P., Tiilikainen A., Selroos O., Kuitunen P. (1976). Family studies in dermatitis herpetiformis. Ann. Clin. Res..

[17] Katz S.I., Falchuk Z.M., Dahl M.V., Rogentine G.N., Strober W. (1972). HL-A8: A genetic link between dermatitis herpetiformis and gluten-sensitive enteropathy. J. Clin. Investig..

[18] Spurkland A., Ingvarsson G., Falk E.S., Knutsen I., Sollid L.M., Thorsby E. (1997). Dermatitis herpetiformis and celiac disease are both primarily associated with the HLA-DQ (alpha 1*0501, beta 1*02) or the HLA-DQ (alpha 1*03, beta 1*0302) heterodimers. Tissue Antigens.

[19] Lemberg D., Day A.S., Bohane T. (2005). Coeliac disease presenting as dermatitis herpetiformis in infancy. J. Paediatr. Child Health.

[20] Ermacora E., Prampolini L., Tribbia G., Pezzoli G., Gelmetti C., Cucchi G., Tettamanti A., Giunta A., Gianotti F. (1986). Long-term follow-up of dermatitis herpetiformis in children. J. Am. Acad. Dermatol..

[21] Smith J.B., Tulloch J.E., Meyer L.J., Zone J.J. (1992). The incidence and prevalence of dermatitis herpetiformis in Utah. Arch. Dermatol..

[22] Salmi T.T., Hervonen K., Kautiainen H., Collin P., Reunala T. (2011). Prevalence and incidence of dermatitis herpetiformis: A 40-year prospective study from Finland. Br. J. Dermatol..

[23] West J., Fleming K.M., Tata L.J., Card T.R., Crooks C.J. (2014). Incidence and prevalence of celiac disease and dermatitis herpetiformis in the UK over two decades: Population-based study. Am. J. Gastroenterol..

[24] Reunala T., Lokki J. (1978). Dermatitis herpetiformis in Finland. Acta Derm. Venereol..

[25] Antiga E., Verdelli A., Calabrò A., Fabbri P., Caproni M. (2013). Clinical and immunopathological features of 159 patients with dermatitis herpetiformis: An Italian experience. G. Ital. Dermatol. Venereol..

[26] Reunala T., Hervonen K., Salmi T. (2021). Dermatitis Herpetiformis: An Update on Diagnosis and Management. Am. J. Clin. Dermatol..

[27] McGovern T.W., Bennion S.D. (1994). Palmar purpura: An atypical presentation of childhood dermatitis herpetiformis. Pediatr. Dermatol..

[28] Karpati S., Torok E., Kosnai I. (1986). Discrete palmar and plantar symptoms in children with dermatitis herpetiformis Duhring. Cutis.

[29] López Aventín D., Ilzarbe L., Herrero-González J.E. (2013). Recurrent digital petechiae and weight loss in a young adult. Gastroenterology.

[30] Savilahti E., Reunala T., Mäki M. (1992). Increase of lymphocytes bearing the gamma/delta T cell receptor in the jejunum of patients with dermatitis herpetiformis. Gut.

[31] Mansikka E., Hervonen K., Kaukinen K., Collin P., Huhtala H., Reunala T., Salmi T. (2018). Prognosis of Dermatitis Herpetiformis Patients with and without Villous Atrophy at Diagnosis. Nutrients.

[32] Leffler D.A., Green P.H., Fasano A. (2015). Extraintestinal manifestations of coeliac disease. Nat. Rev. Gastroenterol. Hepatol..

[33] Ashtari S., Pourhoseingholi M.A., Rostami K., Aghdaei H.A., Rostami-Nejad M., Busani L., Tavirani M.R., Zali M.R. (2019). Prevalence of gluten-related disorders in Asia-Pacific region: A systematic review. J. Gastrointestin. Liver Dis..

[34] Pasternack C., Kaukinen K., Kurppa K., Mäki M., Collin P., Hervonen K., Reunala T., Huhtala H., Kekkonen L., Salmi T. (2017). Gastrointestinal Symptoms Increase the Burden of Illness in Dermatitis Herpetiformis: A Prospective Study. Acta Derm. Venereol..

[35] Kurppa K., Koskinen O., Collin P., Mäki M., Reunala T., Kaukinen K. (2008). Changing phenotype of celiac disease after long-term gluten exposure. J. Pediatr. Gastroenterol. Nutr..

[36] Karell K., Korponay-Szabo I., Szalai Z., Holopainen P., Mustalahti K., Collin P., Mäki M., Partanen J. (2002). Genetic dissection between coeliac disease and dermatitis herpetiformis in sib pairs. Ann. Hum. Genet..

[37] Bolotin D., Petronic-Rosic V. (2011). Dermatitis herpetiformis. Part II. Diagnosis, management, and prognosis. J. Am. Acad. Dermatol..

[38] Hietikko M., Hervonen K., Salmi T., Ilus T., Zone J.J., Kaukinen K., Reunala T., Lindfors K. (2018). Disappearance of epidermal transglutaminase and IgA deposits from the papillary dermis of patients with dermatitis herpetiformis after a long-term gluten-free diet. Br. J. Dermatol..

[39] Pierard J., Whimster I. (1961). The histological diagnosis of dermatitis herpetiformis, bullous pemphigoid and erythema multifore. Br. J. Dermatol..

[40] Bresler S.C., Granter S.R. (2015). Utility of direct immunofluorescence testing for IgA in patients with high and low clinical suspicion for dermatitis herpetiformis. Am. J. Clin. Pathol..

[41] Schultz B., Hook K. (2019). Bullous Diseases in Children: A Review of Clinical Features and Treatment Options. Paediatr. Drugs.

[42] Dieterich W., Laag E., Bruckner-Tuderman L., Reunala T., Kárpáti S., Zágoni T., Riecken E.O., Schuppan D. (1999). Antibodies to tissue transglutaminase as serologic markers in patients with dermatitis herpetiformis. J. Investig. Dermatol..

[43] Sárdy M., Kárpáti S., Merkl B., Paulsson M., Smyth N. (2002). Epidermal transglutaminase (TGase 3) is the autoantigen of dermatitis herpetiformis. J. Exp. Med..

[44] Zhu Y.I., Stiller M.J. (2001). Dapsone and sulfones in dermatology: Overview and update. J. Am. Acad. Dermatol..

[45] Boehncke W.H., Schon M.P. (2015). Psoriasis. Lancet.

[46] Rachakonda T., Schupp C., Armstrong A. (2014). Psoriasis prevalence among adults in the United States. J. Am. Acad. Dermatol..

[47] Bronckers I.M., Paller A.S., van Geel M.J., van de Kerkhof P.C., Seyger M.M. (2015). Psoriasis in Children and Adolescents: Diagnosis, Management and Comorbidities. Paediatr. Drugs.

[48] Tollefson M.M., Crowson C.S., McEvoy M.T., Maradit Kremers H. (2010). Incidence of psoriasis in children: A population-based study. J. Am. Acad. Dermatol..

[49] Kumar B., Jain R., Sandhu K., Kaur I., Handa S. (2004). Epidemiology of childhood psoriasis: A study of 419 patients from northern India. Int. J. Dermatol..

[50] Seyhan M., Coskun B.K., Saglam H., Ozcan H., Karincaoglu Y. (2006). Psoriasis in childhood and adolescence: Evaluation of demographic and clinical features. Pediatr. Int..

[51] Eichenfield L.F., Paller A.S., Tom W.L., Sugarman J., Hebert A.A., Friedlander S.F., Siegfried E., Silverberg N., Cordoro K.M. (2018). Pediatric psoriasis: Evolving perspectives. Pediatr. Dermatol..

[52] Birkenfeld S., Dreiher J., Weitzman D., Cohen A.D. (2009). Coeliac disease associated with psoriasis. Br. J. Dermatol..

[53] Ojetti V., Aguilar Sanchez J., Guerriero C., Fossati B., Capizzi R., De Simone C., Migneco A., Amerio P., Gasbarrini G., Gasbarrini A. (2003). High prevalence of celiac disease in psoriasis. Am. J. Gastroenterol..

[54] Nagui N., El Nabarawy E., Mahgoub D., Mashaly H.M., Saad N.E., El-Deeb D.F. (2011). Estimation of (IgA) anti-gliadin, anti-endomysium and tissue transglutaminase in the serum of patients with psoriasis. Clin. Exp. Dermatol..

[55] Acharya P., Mathur M. (2020). Association between psoriasis and celiac disease: A systematic review and meta-analysis. J. Am. Acad. Dermatol..

[56] Ungprasert P., Wijarnpreecha K., Kittanamongkolchai W. (2017). Psoriasis and Risk of Celiac Disease: A Systematic Review and Meta-analysis. Indian J. Dermatol..

[57] Ludvigsson J.F., Lindelöf B., Zingone F., Ciacci C. (2011). Psoriasis in a nationwide cohort study of patients with celiac disease. J. Investig. Dermatol..

[58] De Vos R., Boer W., Haas F. (1995). Is there a relationship between psoriasis and coeliac disease?. J. Intern. Med..

[59] Zamani F., Alizadeh S., Amiri A., Shakeri R., Robati M., Alimohamadi S.M., Abdi H., Malekzadeh R. (2010). Psoriasis and Coeliac Disease; Is There Any Relationship?. Acta Derm. Venereol..

[60] Kia K., Nair R., Ike R., Hiremagalore R., Elder J., Ellis C. (2007). Prevalence of Antigliadin Antibodies in Patients with Psoriasis is Not Elevated Compared with Controls. Am. J. Clin. Dermatol..

[61] Cardinali C., Degl’innocenti D., Caproni M., Fabbri P. (2002). Is the search for serum antibodies to gliadin, endomysium and tissue transglutaminase meaningful in psoriatic patients? Relationship between the pathogenesis of psoriasis and coeliac disease. Br. J. Dermatol..

[62] Singh S., Sonkar G.K., Singh S. (2010). Celiac disease-associated antibodies in patients with psoriasis and correlation with HLA Cw6. J. Clin. Lab. Anal..

[63] Wu J.J., Nguyen T.U., Poon K.Y., Herrinton L.J. (2012). The association of psoriasis with autoimmune diseases. J. Am. Acad. Dermatol..

[64] Assa A., Frenkel-Nir Y., Tzur D., Katz L.H., Shamir R. (2017). Large population study shows that adolescents with celiac disease have an increased risk of multiple autoimmune and nonautoimmune comorbidities. Acta Paediatr..

[65] Aletaha D., Epstein A.J., Skup M., Zueger P., Garg V., Panaccione R. (2019). Risk of Developing Additional Immune-Mediated Manifestations: A Retrospective Matched Cohort Study. Adv. Ther..

[66] Osier E., Wang A.S., Tollefson M.M., Cordoro K.M., Daniels S.R., Eichenfield A., Gelfand J.M., Gottlieb A.B., Kimball A.B., Lebwohl M. (2017). Pediatric Psoriasis Comorbidity Screening Guidelines. JAMA Dermatol..

[67] Michaëlsson G., Gerdén B., Hagforsen E., Nilsson B., Pihl-Lundin I., Kraaz W., Hjelmquist G., Lööf L. (2000). Psoriasis patients with antibodies to gliadin can be improved by a gluten-free diet. Br. J. Dermatol..

[68] Kolchak N.A., Tetarnikova M.K., Theodoropoulou M.S., Michalopoulou A.P., Theodoropoulos D.S. (2018). Prevalence of antigliadin IgA antibodies in psoriasis vulgaris and response of seropositive patients to a gluten-free diet. J. Multidiscip. Healthc..

[69] De Bastiani R., Gabrielli M., Lora L., Napoli L., Tosetti C., Pirrotta E., Ubaldi E., Bertolusso L., Zamparella M., De Polo M. (2015). Association between coeliac disease and psoriasis: Italian primary care multicentre study. Dermatology.

[70] Addolorato G., Parente A., de Lorenzi G., D’angelo Di Paola M.E., Abenavoli L., Leggio L., Capristo E., De Simone C., Rotoli M., Rapaccini G.L. (2003). Rapid regression of psoriasis in a coeliac patient after gluten-free diet. A case report and review of the literature. Digestion.

[71] Komarova O.N., Khavkin A.I. (2016). Coeliac disease and psoriasis combination in 5-year-old child. Eksp. Klin. Gastroenterol..

[72] Lee H.H., Gwillim E., Patel K.R., Hua T., Rastogi S., Ibler E., Silverberg J.I. (2020). Epidemiology of alopecia areata, ophiasis, totalis, and universalis: A systematic review and meta-analysis. J. Am. Acad. Dermatol..

[73] Gilhar A., Etzioni A., Paus R. (2012). Alopecia areata. N. Engl. J. Med..

[74] Strazzulla L., Wang E.H.C., Avila L., Lo Sicco K., Brinster N., Christiano A.M., Shapiro J. (2018). Alopecia areata: Disease characteristics, clinical evaluation, and new perspectives on pathogenesis. J. Am. Acad. Dermatol..

[75] Gupta M.A., Gupta A.K., Watteel G.N. (1997). Stress and alopecia areata: A psychodermatologic study. Acta Derm. Venereol..

[76] Messenger A.G., McKillop J., Farrant P., McDonagh A.J., Sladden M. (2012). British Association of Dermatologists’ guidelines for the management of alopecia areata 2012. Br. J. Dermatol..

[77] Safavi K.H., Muller S.A., Suman V.J., Moshell A.N., Melton L.J. (1995). Incidence of alopecia areata in Olmsted County, Minnesota, 1975 through 1989. Mayo Clin. Proc..

[78] Uzuncakmak T.K., Engin B., Serdaroglu S., Tuzun Y. (2021). Demographic and Clinical Features of 1641 Patients with Alopecia Areata, Alopecia Totalis, and Alopecia Universalis: A Single-Center Retrospective Study. Skin. Appendage Disord..

[79] De Waard-van der Spek F.B., Oranje A.P., De Raeymaecker D.M., Peereboom-Wynia J.D. (1989). Juvenile versus maturity-onset alopecia areata--a comparative retrospective clinical study. Clin. Exp. Dermatol..

[80] Chelidze K., Lipner S.R. (2018). Nail changes in alopecia areata: An update and review. Int. J. Dermatol..

[81] Alkhalifah A., Alsantali A., Wang E., McElwee K.J., Shapiro J. (2010). Alopecia areata update: Part I. Clinical picture, histopathology, and pathogenesis. J. Am. Acad. Dermatol..

[82] Xing L., Dai Z., Jabbari A., Cerise J.E., Higgins C.A., Gong W., de Jong A., Harel S., DeStefano G.M., Rothman L. (2014). Alopecia areata is driven by cytotoxic T lymphocytes and is reversed by JAK inhibition. Nat. Med..

[83] Ertekin V., Selimoglu M.A., Altinkaynak S. (2009). Celiac disease in childhood: Evaluation of 140 patients. Eurasian J. Med..

[84] Guariso G., Conte S., Presotto F., Basso D., Brotto F., Visonà Dalla Pozza L., Pedini B., Betterle C. (2007). Clinical, subclinical and potential autoimmune diseases in an Italian population of children with coeliac disease. Aliment. Pharmacol. Ther..

[85] Corazza G.R., Andreani M.L., Venturo N., Bernardi M., Tosti A., Gasbarrini G. (1995). Celiac disease and alopecia areata: Report of a new association. Gastroenterology.

[86] Volta U., Bardazzi F., Zauli D., DeFranceschi L., Tosti A., Molinaro N., Ghetti S., Tetta C., Grassi A., Bianchi F.B. (1997). Serological screening for coeliac disease in vitiligo and alopecia areata. Br. J. Dermatol..

[87] Mokhtari F., Panjehpour T., Naeini F.F., Hosseini S.M., Nilforoushzadeh M.A., Matin M. (2016). The Frequency Distribution of Celiac Autoantibodies in Alopecia Areata. Int. J. Prev. Med..

[88] Pham C.T., Romero K., Almohanna H.M., Griggs J., Ahmed A., Tosti A. (2020). The Role of Diet as an Adjuvant Treatment in Scarring and Nonscarring Alopecia. Skin Appendage Disord..

[89] Barbato M., Viola F., Grillo R., Franchin L., Lo Russo L., Lucarelli S., Frediani T., Mazzilli M.C., Cardi E. (1998). Alopecia and coeliac disease: Report of two patients showing response to gluten-free diet. Clin. Exp. Dermatol..

[90] Fessatou S., Kostaki M., Karpathios T. (2003). Coeliac disease and alopecia areata in childhood. J. Paediatr. Child Health.

[91] Bardella M.T., Marino R., Barbareschi M., Bianchi F., Faglia G., Bianchi P. (2000). Alopecia areata and coeliac disease: No effect of a gluten-free diet on hair growth. Dermatology.

[92] Bondavalli P., Quadri G., Parodi A., Rebora A. (1998). Failure of gluten-free diet in celiac disease associated alopecia areata. Acta Derm. Venereol..

[93] Radonjic-Hoesli S., Hofmeier K.S., Micaletto S., Schmid-Grendelmeier P., Bircher A., Simon D. (2018). Urticaria and Angioedema: An Update on Classification and Pathogenesis. Clin. Rev. Allergy Immunol..

[94] Zuberbier T., Aberer W., Asero R., Bindslev-Jensen C., Abdul Latiff A.H., Baker D., Ballmer-Weber B., Bernstein J.A., Bindslev-Jensen C., Brzoza Z. (2014). The EAACI/GA2LEN/EDF/WAO Guideline for the definition, classification, diagnosis, and management of urticaria: The 2013 revision and update. Allergy.

[95] Fine L.M., Bernstein J.A. (2016). Guideline of chronic urticaria beyond. Allergy Asthma Immunol. Res..

[96] O’Donnell B., Lawlor F., Simpson J., Morgan M., Greaves M. (1997). The impact of chronic urticaria on the quality of life. Br. J. Dermatol..

[97] Hauteke M., De Clerck L., Stevens W. (1987). Chronic urticaria associated with coeliac disease. Lancet.

[98] Caminiti L., Passalacqua G., Magazzu G., Comisi F., Vita D., Barberio G., Sferlazzas C., Pajno G.B. (2005). Chronic urticaria and associated coeliac disease in children: A case-control study. Pediatr. Allergy Immunol..

[99] Ludvigsson J.F., Lindelöf B., Rashtak S., Rubio-Tapia A., Murray J.A. (2013). Does urticaria risk increase in patients with celiac disease? A large population-based cohort study. Eur. J. Dermatol..

[100] Levine A., Dalal I., Bujanover Y. (1999). Celiac disease associated with familial chronic urticaria and thyroid autoimmunity in a child. Pediatrics.

[101] Abenavoli L., Proietti L., Leggio L., Ferrulli A., Vonghia L., Capizzi R., Rotoli M., Amerio P.L., Gasbarrini G., Addolorato G. (2006). Cutaneous manifestations in celiac disease. World J. Gastroenterol..

[102] Peroni D.G., Paiola G., Tenero L., Fornaro M., Bodini A., Pollini F., Piacentini G.L. (2010). Chronic urticaria and celiac disease: A case report. Pediatr. Dermatol..

[103] Kolkhir P., Borzova E., Grattan C., Asero R., Pogorelov D., Maurer M. (2017). Autoimmune comorbidity in chronic spontaneous urticaria: A systematic review. Autoimmun. Rev..

[104] Greaves M.W. (2003). Chronic idiophatic urticaria. Curr. Opin. Allergy Clin. Immunol..

[105] Nutten S. (2015). Atopic dermatitis: Global epidemiology and risk factors. Ann. Nutr. Metabl..

[106] Darlenski R., Kazandjieva J., Hristakieva E., Fluhr J.W. (2014). Atopic dermatitis as a systemic disease. Clin. Dermatol..

[107] Spergel J.M. (2010). From atopic dermatitis to asthma: The atopic march. Ann. Allergy Asthma Immunol..

[108] Smidesang I., Saunes M., Storrø O., Øien T., Holmen T.L., Johnsen R., Henriksen A.H. (2008). Atopic dermatitis among 2-year olds; high prevalence, but predominantly mild disease--the PACT study, Norway. Pediatr. Dermatol..

[109] De Bruin Weller M.S., Knulst A.C., Meijer Y., Bruijnzeel-Koomen C.A.F.M., Pasmans S.G.M. (2012). Evaluation of the child with atopic dermatitis. Clin. Exp. Allergy.

[110] Narla S., Silverberg J.I. (2019). Association between atopic dermatitis and autoimmune disorders in US adults and children: A cross-sectional study. J. Am. Acad. Dermatol..

[111] Verkasalo M., Tiilikainen A., Kuitunen P., Savilahti E., Backman A. (1983). HLA antigens and atopy in children with coeliac disease. Gut.

[112] Greco L., De Seta L., D’Adamo G., Baldassarre C., Mayer M., Siani P., Lojodice D. (1990). Atopy and coeliac disease: Bias or true relation?. Acta Paediatr. Scand..

[113] Ress K., Annus T., Putnik U., Luts K., Uibo R., Uibo O. (2014). Celiac disease in children with atopic dermatitis. Pediatr. Dermatol..

[114] Shalom G., Kridin K., Raviv K.O., Freud T., Comaneshter D., Friedland R., Cohen A.D., Ben-Amitai D. (2020). Atopic Dermatitis and Celiac Disease: A Cross-Sectional Study of 116,816 Patients. Am. J. Clin. Dermatol..

[115] Kauppi S., Jokelainen J., Timonen M., Tasanen K., Huilaja L. (2021). Atopic Dermatitis Is Associated with Dermatitis Herpetiformis and Celiac Disease in Children. J. Investig. Dermatol..

[116] Bowen T., Cicardi M., Bork K., Zuraw B., Frank M., Ritchie B., Farkas H., Varga L., Zingale L.C., Binkley K. (2008). Hereditary angiodema: A current state-of-the-art review, VII: Canadian Hungarian 2007 International Consensus Algorithm for the Diagnosis, Therapy, and Management of Hereditary Angioedema. Ann. Allergy Asthma Immunol..

[117] Farkas H., Visy B., Fekete B., Karádi I., Kovács J.B., Kovács I.B., Kalmár L., Tordai A., Varga L. (2002). Association of celiac disease and hereditary angioneurotic edema. Am. J. Gastroenterol..

[118] Csuka D., Kelemen Z., Czaller I., Molnár K., Füst G., Varga L., Rajczy K., Szabó Z., Miklós K., Bors A. (2011). Association of celiac disease and hereditary angioedema due to C1-inhibitor deficiency. Screening patients with hereditary angioedema for celiac disease: Is it worth the effort?. Eur. J. Gastroenterol. Hepatol..

[119] Similä S., Kokkonen J., Kallioinen M. (1982). Cutaneous vasculitis as a manifestation of coeliac disease. Acta Paediatr. Scand..

[120] Şahin Y., Şahin S., Adrovic A., Kutlu T., Çokuğras F.Ç., Barut K., Erkan T., Kasapçopur Ö. (2019). Serological screening for celiac disease in children with systemic lupus erythematosus. Eur. J. Rheumatol..

[121] Alves S.C., Fasano S., Isenberg D.A. (2016). Autoimmune gastrointestinal complications in patients with systemic lupus erythematosus: Case series and literature review. Lupus.

[122] Crişcov G.I., Stana A.B., Ioniue I.K., Alexoae M.M., Moraru E. (2015). Coexistence of celiac disease and systemic lupus erythematosus in a 6-year-old girl-case report. Rev. Med. Chir. Soc. Med. Nat. Iasi.

[123] Hadjivassilious M., Sanders D.S., Grünewald R.A., Akil M. (2004). Gluten sensitivity masquerading as systemic lupus erythematosus. Ann. Rheum. Dis..

[124] Ergül B., Koçak E., Köklü S. (2012). Behcet disease and celiac disease: To screen or not?. Rheumatol. Int..

[125] Buderus S., Wagner N., Lentze M.J. (1997). Concurrence of celiac disease and juvenile dermatomyositis: Result of a specific immunogenetic susceptibility?. J. Pediatr. Gastroenterol. Nutr..

[126] Molnár K., Torma K., Siklós K., Csanády K., Korponay-Szabó I., Szalai Z. (2006). Juvenile dermatomyositis and celiac disease. A rare association. Eur. J. Pediatr. Dermatol..

[127] Abenavoli L., Dastoli S., Bennardo L., Boccuto L., Passante M., Silvestri M., Proietti I., Potenza C., Luzza F., Nisticò S.P. (2019). The Skin in Celiac Disease Patients: The Other Side of the Coin. Medicina.

[128] Zone J.J. (2005). Skin manifestations of celiac disease. Gastroenterology.

[129] Bartyik K., Várkonyi A., Kirschner A., Endreffy E., Túri S., Karg E. (2004). Erythema nodosum in association with celiac disease. Pediatr. Dermatol..

[130] Vaz S.O., Franco C., Santos P., Amaral R. (2018). Skin and coeliac disease, a lot to think about: A case series. BMJ Case Rep..

[131] Troncone R., Discepolo V. (2014). Celiac disease and autoimmunity. J. Pediatr. Gastroenterol. Nutr..

[132] Nunnemann S., Uibel C., Budig P., Mäurer M. (2020). Acute intermittent porphyria (AIP) in a patient with celiac disease. Neurol. Res. Pract..

[133] Nenna R., D’Eufemia P., Celli M., Mennini M., Petrarca L., Zambrano A., Montuori M., La Pietra M., Bonamico M. (2011). Celiac disease and lamellar ichthyosis. Case study analysis and review of the literature. Acta Dermatovenerol. Croat..

[134] Howard G., Horev A., Samueli B., Yerushalmi B. (2021). Morphea as Part of the Dermatological Manifestation of Celiac Disease: Case Presentation and Review of the Literature. Case Rep. Dermatol..

[135] Woolfson H., McQueen A., Stephen M. (1974). Erythroderma in a child with coeliac disease. Br. J. Dermatol..

[136] Thelander H.E. (1946). Leiner’s disease followed by the celiac syndrome; a case report. J. Pediatr..

[137] Garcia Y.H., Díez S.G., Aizpún L.T., Oliva N.P. (2002). Antigliadin antibodies associated with chronic mucocutaneous candidiasis. Pediatr. Dermatol..

[138] Brinkert F., Sornsakrin M., Krebs-Schmitt D., Ganschow R. (2009). Chronic mucocutaneous candidiasis may cause elevated gliadin antibodies. Acta Paediatr..

[139] Müller S., Beissert S., Metze D., Luger T.A., Bonsmann G. (1999). Lipodystrophia centrifugalis abdominalis infantilis in a 4-year-old Caucasian girl: Association with partial IgA deficiency and autoantibodies. Br. J. Dermatol..

[140] Yılmaz S., Tuna Kırsaçlıoğlu C., Şaylı T.R. (2020). Celiac disease and hematological abnormalities in children with recurrent aphthous stomatitis. Pediatr. Int..

[141] Campisi G., Di Liberto C., Carroccio A., Compilato D., Iacono G., Procaccini M., Di Fede G., Lo Muzio L., Craxi A., Catassi C. (2008). Coeliac disease: Oral ulcer prevalence, assessment of risk and association with gluten-free diet in children. Dig. Liver Dis..

[142] Mishra P., Sirka C.S., Das R.R., Nanda D. (2016). Secondary acrodermatitis enteropathica-like lesions in a child with newly diagnosed coeliac disease. Paediatr. Int. Child Health.

[143] Macho V.M.P., Coelho A.S., Veloso E., Silva D.M., de Andrade D.J.C. (2017). Oral Manifestations in Pediatric Patients with Coeliac Disease—A Review Article. Open Dent. J..

